# QTL mapping and stability analysis of trichome density in zucchini (*Cucurbita pepo* L.)

**DOI:** 10.3389/fpls.2023.1232154

**Published:** 2023-08-11

**Authors:** Yunli Wang, Guichao Wang, Dongjuan Lin, Qinfen Luo, Wenlong Xu, Shuping Qu

**Affiliations:** ^1^ Key Laboratory of Biology and Genetic Improvement of Horticultural Crops (Northeast Region), Ministry of Agriculture and Rural Affairs/Northeast Agricultural University, Harbin, China; ^2^ College of Horticulture and Landscape Architecture, Northeast Agricultural University, Harbin, China

**Keywords:** *Cucurbita pepo*, trichome density, InDel markers, QTL analysis, zinc finger protein

## Abstract

Trichomes provide an excellent model for studying cell differentiation and proliferation. The aboveground tissues of plants with long dense trichomes (LDTs) can cause skin itching in people working in a zucchini field, in which management, pollination, and fruit harvesting are difficult. In this study, an F_2_ population was constructed with the LDT inbred line “16” and the sparse micro trichome (SMT) inbred line “63” for QTL analysis of type I and II trichome density. Two QTLs were identified on chromosomes 3 and 15 using the QTL-seq method. Additionally, 191 InDel markers were developed on 20 chromosomes, a genetic map was constructed for QTL mapping, and three QTLs were identified on chromosomes 3, 6, and 15. Two QTLs, *CpTD3.1* and *CpTD15.1*, were identified in both QTL-seq and genetic map-based QTL analyses, and *CpTD15.1* was the major-effect QTL. The stability of *CpTD3.1* and *CpTD15.1* was confirmed using data from F_2_ plants under different environmental conditions. The major-effect QTL *CpTD15.1* was located between markers chr15-4991349 and chr15-5766791, with a physical distance of 775.44 kb, and explained 12.71%–29.37% of the phenotypic variation observed in the three environments. *CpTD3.1* was located between markers chr3-218350 and chr3-2891236, in a region with a physical distance of 2,672.89 kb, and explained 5.00%–10.64% of the phenotypic variation observed in the three environments. The functional annotations of the genes within the *CpTD15.1* region were predicted, and five genes encoding transcription factors regulating trichome development were selected. *Cp4.1LG15g04400* encoded zinc finger protein (ZFP) and harbored nonsynonymous SNPs in the conserved ring finger domain between the two parental lines. There were significant differences in *Cp4.1LG15g04400* expression between “16” and “63”, and a similar pattern was found between germplasm resources of LDT lines and SMT lines. It was presumed that *Cp4.1LG15g04400* might regulate trichome density in zucchini. These results lay a foundation for better understanding the density of multicellular nonglandular trichomes and the regulatory mechanism of trichome density in zucchini.

## Introduction

*Cucurbita pepo* L. is one of the cultivated *Cucurbita* spp. and has the most abundant germplasm resources among *Cucurbita* crops. Zucchini not only shows strong stress resistance and adaptability, but also had high nutritional, medicinal, and ornamental value. Therefore, zucchini is grown widely from tropical to temperate areas. The surfaces of the aboveground tissues of most zucchini germplasm resources are covered with sharp dense trichomes. During the field production of zucchini, the sharp dense trichomes of zucchini plants can cause skin itchiness in people managing or pollinating plants or harvesting their fruit. Furthermore, the dense trichomes of fruit stems easily scratch young zucchini fruits, which is unfavorable for fruit transport. Therefore, it is necessary to identify and clone genes controlling trichome density trait in zucchini.

Trichomes are the first functional barrier in contact with the outside world and play a significant role in plant development and the biotic and abiotic adaptations, and some of them have important commercial value (Amme et al., 2005; Emiko et al., 2010; An et al., 2012). Trichomes are widely distributed on the surfaces of the aboveground parts of land plants, such as leaves, petioles, stems, pericarps, fruits, tendrils, and pedicels (Liu et al., 2016). Different arrangements and numbers of trichome cells develop into different morphologies, and according to trichome morphology, plant trichomes are divided into unicellular or multicellular trichomes (Schnittger et al., 1999). Unicellular trichomes are nonglandular, and some have branches, while multicellular trichomes are divided into glandular or nonglandular trichomes (Schnittger et al., 1999). Common multicellular trichomes had complex structures and consist of three components: head cells, stalk cells, and basal cells (Amme et al., 2005; Chang et al., 2016).

Trichomes in *Arabidopsis* are typically unicellular branched structures. Numerous genes have been identified as key regulators of trichome initiation. The regulatory network affecting trichome initiation and development was constructed, and approximately 40 genes were involved. In *Arabidopsis*, the R2R3 MYB transcription factor GLABRA 1 (GL1) (Kirik et al., 2004), the basic helix-loop-helix (bHLH) transcription factor GLABRA 3/ENHANCER OF GLABRA 3 (GL3/EGL3), and the pleiotropic WD40 repeat protein TRANSPARENT TESTA GLABRA 1 (TTG1) (Walker et al., 1999; Zhao et al., 2008) formed the MYB-bHLH-WDR (MBW) complex, which was the key core of the trichome initiation pathway. The MBW complex activates trichome formation by enhancing the expression of downstream homeodomain-leucine zipper (HD-ZIP) IV genes, *GL2* and *EGL2* (Payne et al., 2000; Ishida et al., 2007). R3-type MYB transcription factors CAPRICE (CPC), ENHANCER OF TRY AND CPC 1 (ETC1), ETC2, ETC3, TRIPTYCHON (TRY), TRICHOMELESS 1 (TCL1), and TCL2 act as negative regulators of trichome formation by competing with *GL1* and interacting with *GL3*/*EGL3* to form an inactive complex (Schnittger et al., 1999; Kirik et al., 2004; Wang et al., 2007; Wang et al., 2008; Wester et al., 2009). bHLH transcription factor MYC1 positively regulates trichome number (Zhao et al., 2012). C2H2 type zinc-finger proteins (ZFPs), such as glabrous inflorescence stems (GIS), GIS2, GIS3, ZFP5, ZFP6, and ZFP8, play key roles in the initiation and formation of leaf trichomes (Gan et al., 2007; Zhou et al., 2012; Zhou et al., 2013; Sun et al., 2015; Liu et al., 2017; Kim et al., 2018). The AP2/ERF family transcription factor TARGET OF EARLY ACTIVATION TAGGED 1 (TOE1) regulates trichome initiation of the main-stem inflorescence (Liu et al., 2023). SET Domain Group 26 (SDG26), a histone lysine methyltransferase, affects trichome growth and development (Zeng et al., 2023).

Tomato trichomes have unbranched multicellular structures and are divided into eight types. Types I, IV, VI, and VII are glandular types with secretory capacity, whereas types II, III, V, and VIII are nonglandular types (Amme et al., 2005). The functions of an HD-ZIP IV family gene, *Woolly* (*Wo*), are involved in the formation and development of type I and IV trichomes (Yang et al., 2011). A B-type cyclin gene, *SlCycB2*, participates in the development of type I, III, and V trichomes (Gao et al., 2017). *SlMYC1*, a bHLH transcription factor, regulates not only the development of type VI trichomes but also mono- and sesquiterpene biosynthesis in tomato (Xu et al., 2018). An R2R3-MYB family gene, *SlMIXTA*, functions as a positive regulator of tomato trichome formation (Ying et al., 2020). SlHair (SlH), a C2H2 zinc finger protein (ZFP), positively regulates the number and length of type I trichomes in tomato (Chun et al., 2021).

Cucumber fruit is covered with spines (a special type of trichome) and tubercules, which are important agronomic traits that affect commercial value. Fruit spines have branchless multicellular structures. Fruit spines are divided into eight types: types I and VI are glandular and types II, III, IV, V, VII, and VIII are nonglandular (Xue et al., 2019). The HD-ZIP I transcription factor genes *Glabrous 1*/*Tny branched hair*/*Micro-trichome* (*CsGL1*/*TBH*/*MICT*) are key allelic genes involved in spine development and affect spine distribution in cucumber (Chen et al., 2014; Li et al., 2015; Zhao et al., 2015). The HD-ZIP IV transcription factor genes *Glabrous 3/Trichome-less/Few spine 1* (*CsGL3/TRIL/FS1*) are key allelic genes for the positive regulation of cucumber fruit spines’ differentiation, initiation, and development (Liu et al., 2016; Wang et al., 2016; Zhang et al., 2016; Zhang et al., 2019). The C2H2-type zinc finger gene *tuberculate* (*Tu*) is a key gene that controls the formation of tuberculate fruit (Yang et al., 2014). The WD-repeat homologues CsTTG1 and MIXTA-LIKE transcription factor CsMYB6 are key factors involved in regulating the differentiation of fruit spines and the tubercule formation (Chen et al., 2014; Chen et al., 2016; Zhao et al., 2020). bHLH transcription factors, such as CsMYC2, CsMYC4, CsMYC5, CsMYC6, CsMYC7, and CsMYC8, play an important role in glandular trichome development (Feng et al., 2023). In addition to transcription factor genes, other genes are also involved in cucumber spine development. NUMEROUS SPINES (NS) acts as a regulatory factor in spine development by modulating the auxin signaling pathway (Xie et al., 2018). The *tender spines* (*TS*) gene encodes a C-type lectin receptor-like tyrosine-protein kinase and plays an important role in the formation of cucumber spines (Guo et al., 2018).

Gene mapping studies and analyses of regulatory mechanisms related to trichome/spine initiation and development are mainly conducted in model plants, such as *Arabidopsis*, tomato, and cucumber. In a previous report, zucchini trichomes were classified into seven types according to the morphology: type I, II, III, and VII trichomes were nonglandular, and type IV, V, and VI trichomes were glandular. Among them, type I and II trichomes were the longest. The zucchini germplasm resources were divided into a long dense trichome (LDT) group and a sparse micro trichome (SMT) group according to the presence of type I and II trichomes. An F_2_ population derived by crossing the LDT line “16” and SMT line “63” was constructed, and the trichome density exhibited characteristic quantitative inheritance (Lin et al., 2023). Although there have been reports of trichome characteristics, classification, and inheritance, the key genes and regulatory mechanisms related to zucchini trichome development remain to be reported. In our study, quantitative trait loci (QTLs) related to the trichome density were identified using the QTL sequencing (QTL-seq) method and genetic map-based QTL analysis. Additional F_2_ populations grown under different environmental conditions were used to validate the stability of QTLs, and the candidate genes in the major-effect QTL region associated with trichome development were identified. A C3DHC3-type ZFP gene might regulate trichome density in zucchini. The results lay a foundation for better understanding of multicellular trichome development and improving trichome-related breeding in zucchini.

## Materials and methods

### Plant material

The high-generation inbred line “16” showed typical LDT, and type I, II, III, and IV trichomes were distributed on the surfaces of these plants. The high-generation inbred line “63” had SMT, and type III, IV, and VII trichomes were distributed on the surfaces of these plants. For the QTL analysis of trichome density of type I and type II, the F_2_ population was derived from a cross between “16” and “63”. A total of 536 and 238 F_2_ individuals were grown in the spring (S) of 2019 and 2021, respectively. A total of 886 F_2_ individuals were grown in the fall (F) of 2020. Ten “16”, “63”, and F_1_ plants were also investigated under each set of environmental conditions. To investigate the allelic diversity of predicted genes, germplasm resources of five LDT lines (20-23, 21-1, 21-3, 21-6, and 22-4) and five SMT lines (19-7, 19-28, 20-17, 21-9, and 21-15) (15 plants of each line) were planted. Seeds were soaked in water at 55°C for 8 h and then germinated at 28°C. All individuals were planted in a greenhouse at Xiangyang Experimental Agricultural Station of Northeast Agricultural University, Harbin, China (N45°77′, E126°92′). All individuals were subjected to irrigation, hand weeding, and hand pollination.

### Classification of zucchini trichomes

During the growth of the F_2_ population to the flowering stage, the genetic regularity of trichome density was analyzed. Among the aboveground tissues of zucchini, petiole trichomes were the easiest to observe and distinguish. According to the number of trichomes in a 1-mm^2^ area and the mean distance of type I and II trichomes of petioles on the second young leaf, the trichome density phenotypes of individual F_2_ plants were categorized into five classes (Lin et al., 2023). The trichomes of line “16” belonged to class 5, the trichomes of line “63” belonged to class 1, and the trichomes of F_1_ belonged to class 3.

### QTL analysis of trichome density with QTL-seq

Young leaves from the parents and F_1_ and F_2_ populations were collected for DNA extraction using the cetyltrimethylammonium bromide (CTAB) method (Murray and Thompson, 1980). For QTL-seq, a dense trichome pool (D-pool) and a sparse trichome pool (S-pool) were constructed by mixing equal amounts of DNA from 30 dense trichome individuals (class 5) and 30 sparse trichome individuals (class 1) from F_2_. A parental dense trichome pool and a parental sparse trichome pool were constructed by mixing an equal amount of DNA from 20 “16” plants and 20 “63” plants. A paired-end sequencing library with DNA fragments of approximately 350 bp DNA was constructed from the four pools on an Illumina Hi-Seq 2000 sequencer using a commercial service at BioMarker (Peking, China). Reads were mapped to the reference genome of zucchini (http://cucurbitgenomics.org/organism/14, Javier et al., 2018). The output of paired-end alignment files was compared with that of the BWA software (Li and Durbin, 2009) to filter clean reads. SNPs and InDels between “16” and “63”, and between the D-pool and S-pool were identified using the variant analysis software GATK (McKenna et al., 2010). To identify candidate regions associated with trichome density of type I and type II, the SNP/InDel index of Euclidean distance (ED) and the Δ(SNP/InDel index) were calculated for all positions (Abe et al., 2012; Hill et al., 2013). According to the null hypothesis, 99% confidence intervals were selected to identify the candidate regions harboring QTLs for trichome density.

### Genetic map-based analysis of trichome density QTLs

Ninety-three individuals were randomly selected from the F_2_ population in Spring 2021 for genetic map construction and QTL analysis of trichome density. InDel markers on 20 chromosomes were developed based on InDels between the two parental lines using Primer Premier 5.0 (**Table S1**). For InDel markers, PCR was carried out using 10-μl samples containing ~40 ng of genomic DNA, 0.5 μM of each primer, 200 μM dNTPs, 1× reaction buffer, and 0.5 U Taq DNA polymerase (Aidlab Biotechnologies, Beijing, China). PCR amplification was performed with the following PCR program: 94°C for 4 min; 35 cycles of 94°C for 30 s, 57°C for 30 s, and 72°C for 30 s; and 72°C for 10 min. Products were separated on an 8% polyacrylamide gel by electrophoresis (Wang et al., 2020; Wang et al., 2021). After electrophoresis at 220 V for 1.5 h, the gel was stained in 0.2% AgNO_3_ solution and revealed the silver-stained DNA bands (Ding et al., 2021).

A genetic map was constructed with InDel polymorphism markers from the 20 chromosomes using JoinMap 4.0, and recombination values were converted into map distances in centiMorgans (cM) using the Kosambi mapping function. A, B, and H represent homozygous “16”, homozygous “63”, and heterozygous genotypes, respectively. Spearman’s correlation coefficients for each chromosome were calculated using the Statistical Analysis System (SAS) program (ND Times, Peking, China). The marker information in the genetic map and trichome phenotype data were combined for linkage analysis using RStudio (R/qtl) software with the multiple QTL mapping (MQM) method with a walking speed of 1 cM (Weng et al., 2015). The logarithm of odds (LOD) score for a significant QTL was estimated using 1,000 permutations and a *p*-value of 0.05. QTLs that could explain more than 15% of the observed phenotypic variation (PVE) were considered major-effect QTLs. The QTLs were named according to the trait name, chromosome number, and QTL number.

### Stability analysis of QTLs

To confirm the QTLs for trichome density detected by QTL-seq and genetic map-based QTL mapping, we conducted QTL analysis of 536 F_2_ individuals from Spring 2019, 886 individuals from Fall 2020, and 238 individuals from Spring 2021 using InDel markers. InDel markers between the two parental lines on the QTL chromosomes were selected (**Table S2**). The marker information and trichome phenotype data were combined for linkage analysis using RStudio (R/qtl) software via the composite interval mapping (CIM) method with a walking speed of 1 cM (Churchill and Doerge, 1994). The LOD threshold for significant QTL identification was established separately with 1,000 permutation tests (*p* = 0.05) and ranged from 2.88 to 3 for each environment; therefore, an LOD score of 3 was used for QTL detection.

### Sequencing and predictive functions of predicted genes associated with the major-effect QTL

In the major-effect QTL region, the gene IDs, structures, and predicted functions were obtained using online software according to the Cucurbit Genomics Database (Javier et al., 2018). NCBI online software (https://www.ncbi.nlm.nih.gov/) was also used for gene structure identification, conserved domain analysis, and gene function annotation. The coding sequences of full-length candidate genes were sequenced, and primer pairs were designed using Primer 5.0 software.

### RNA isolation and gene expression

The trichomes development of the cotyledon base and leaf primordium was observed, and trichomes from the third stage was the key stage for glandular and nonglandular trichome development (Zhang et al., 2021; Dong et al., 2022). Trichomes from the third stage (the key stage for glandular and nonglandular trichome development) were collected from the base of cotyledon at 5 days after germination and leaf primordium at 7 days after germination on “16” and “63” for trichome analysis at the key developmental stage. Leaf primordium of five LDT lines and five SMT lines were also collected to analyze expression differences between LDT and SMT germplasm resources. RNA was extracted using TRIzol reagent (Invitrogen, USA). Dried RNA samples were dissolved in H_2_O and treated with diethylpyrocarbonate. First-strand cDNA was prepared according to the PrimeScript RT Reagent Kit with the gDNA Eraser (TaKaRa, Kyoto, Japan) protocol. Primer pairs of candidate resistance genes were designed using Primer Premier 5.0 (**Table S3**). Taq SYBR Green qPCR Premix (Yugong Biolabs, Inc., CN) was used to perform qRT-PCR. The program used in this assay was as follows: 96°C for 1 min, 95°C for 15 s, 58°C for 15 s, and 72°C for 45 s, with 35 cycles. The Actin gene was used as the internal reference. Three technical replicates were performed for each sample, and relative expression levels were quantified using the 2^−ΔΔCT^ method (Livak and Schmittgen, 2001).

## Results

### QTL analysis of trichome density with QTL-seq

**Figure 1:**
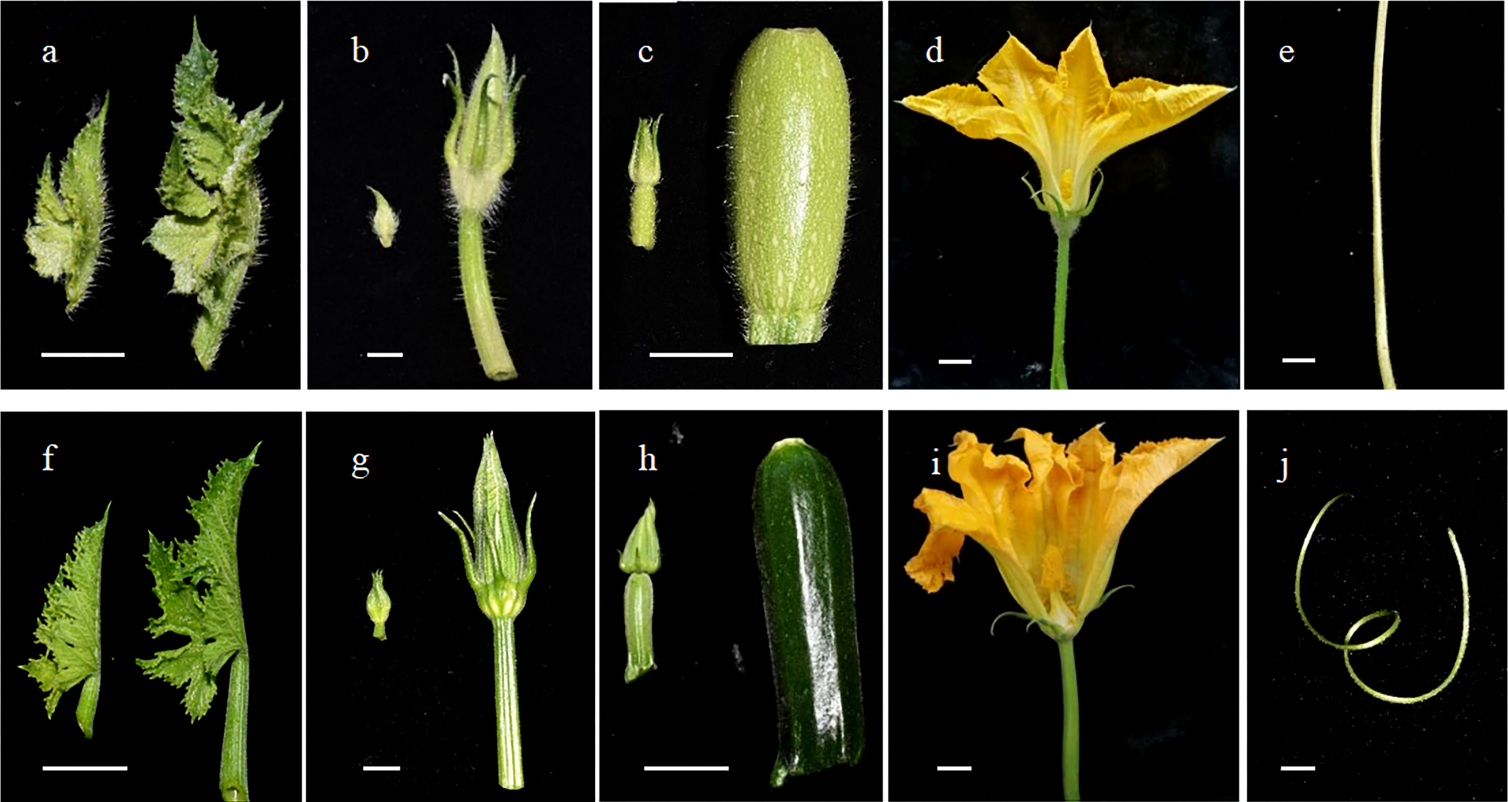
Observation of the trichomes on the surface of tissues. The trichomes on the surface of young leaves **(A)**, flower buds and pedicels **(B)**, ovary and fruit stalks **(C)**, petals and sepals **(D)**, and tendrils **(E)** in “16”. The trichomes on the surface of young leaves **(F)**, flower buds and pedicels **(G)**, ovary and fruit stalks **(H)**, petals and sepals **(I)**, and tendrils **(J)** in “16”. Scale bars represent 1 cm.

The zucchini trichomes were observed to be unbranched multicellular trichomes and classified into seven types according to their trichome structures. Among these types, type I and II trichomes were long and pointy. In “16”, type I and II trichomes were densely arranged on the surface of aboveground tissues, such as leaves, petioles, tendrils, flower buds, petals, fruits, fruit stalks, and pedicels. In “63”, type I and II trichomes were absent on the surface of aboveground tissues (**Figure 1**). In previous studies in our lab, an F_2_ population derived by crossing “16” and “63” was constructed, and the density of type I and type II trichomes exhibited characteristic quantitative inheritance.

DNA pools of “16” and “63”, the D-pool, and the S-pool were sequenced to obtain the candidate regions for trichome density. A total of 47.29 and 38.48 million clean reads with high average read depths (28- and 23-) were obtained from the D- and S-pools, respectively, and the corresponding Q30 values reached 92.42% and 91.49%. The percentages of clean reads from the D- and S-pools that were mapped to the reference genome of zucchini were 98.07% and 97.96%, respectively (**Table S4**). These results indicated that the sequencing results were reliable for QTL-seq. A total of 40,404 high-quality InDels and 80,789 high-quality SNPs were obtained on all 20 chromosomes in zucchini.

**Figure 2:**
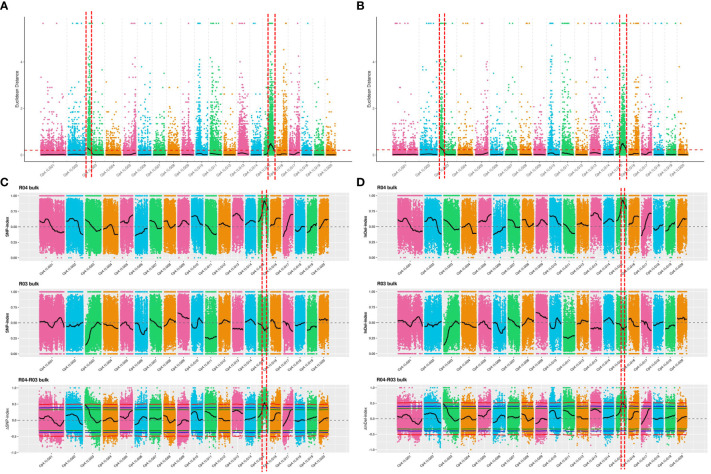
QTL mapping of trichome density by the strategy of QTL-seq combined with linkage analysis. The candidate region from 0 to 3.00 Mb on chromosome 3 and the candidate region from 3.65 Mb to 8.65 Mb on chromosome 15 were obtained. **(A)** Distribution of ED-based linkage value with SNPs on chromosomes. **(B)** Distribution of ED-based linkage value with InDels on chromosomes. **(C)** Distribution of SNP index correlation values on chromosomes. **(D)** Distribution of InDel index correlation values on chromosomes. The *x*-axes indicate chromosomes. In **(A, B)**, Median + 3 SD of the fitted value of all sites were defined as linkage threshold and shown by red lines. In **(C, D)**, the top panel is a distribution map of the SNP index (InDel index) values of the D-pool, the middle panel is a distribution map of the SNP index (InDel index) values of the S-pool, and the bottom panel is a distribution map of the ΔSNP index (ΔInDel index) values. The red line represents the confidence threshold of 0.99, the blue line represents a confidence threshold of 0.95, and the green line represents a confidence threshold of 0.90.

The ED algorithm was employed to identify significantly differential SNPs between the D-pool and S-pool based on sequencing data, to predict candidate regions of trichome density. A 2.70-Mb region and a 5.00-Mb region were identified on chromosomes 3 and 15, respectively (**Figure 2A**). The ED algorithm was employed to identify significantly differential InDels between the D-pool and S-pool, and a 3.00-Mb region and a 4.98-Mb region were identified on chromosomes 3 and 15, respectively (**Figure 2B**). Δ(InDel-index) and Δ(SNP-index) were calculated and plotted by comparing the InDel-index and SNP-index results of the D- and S-pools at the corresponding genomic positions. A 1.11-Mb region of chromosomes 15 was identified based on Δ(InDel-index) values (**Figure 2C**), and a 1.33-Mb region of chromosomes 15 was identified based on Δ(SNP-index) values higher than the threshold value (**Figure 2D**). To obtain all the possible regions associated with trichome density, the convergence of these association regions was considered. The candidate region from 0 to 3.00 Mb on chromosome 3 and the candidate region from 3.65 Mb to 8.65 Mb on chromosome 15 were obtained and found to contain a total of 1,116 genes (**Table S5**).

### QTL analysis of trichome density with genetic mapping

InDel markers on 20 chromosomes were developed based on the InDels identified between the two parental lines, “16” and “63”, for genetic map construction. Finally, a total of 191 polymorphic InDel markers were selected, which were uniformly distributed on 20 chromosomes. The genotypes of the 191 InDel markers in F_2_ individuals are presented in **Table S6**. The genetic map spanned a total of 2,765.65 cM, with an average distance of 14.48 cM between adjacent markers (**Table S7**). The Spearman correlation coefficient of each chromosome ranged from 0.98 to 1 (**Table S7**), which indicated that the order of the InDel markers was highly consistent with their physical locations in zucchini scaffolds. Therefore, this zucchini genetic map was suitable for QTL mapping.

**Figure 3:**
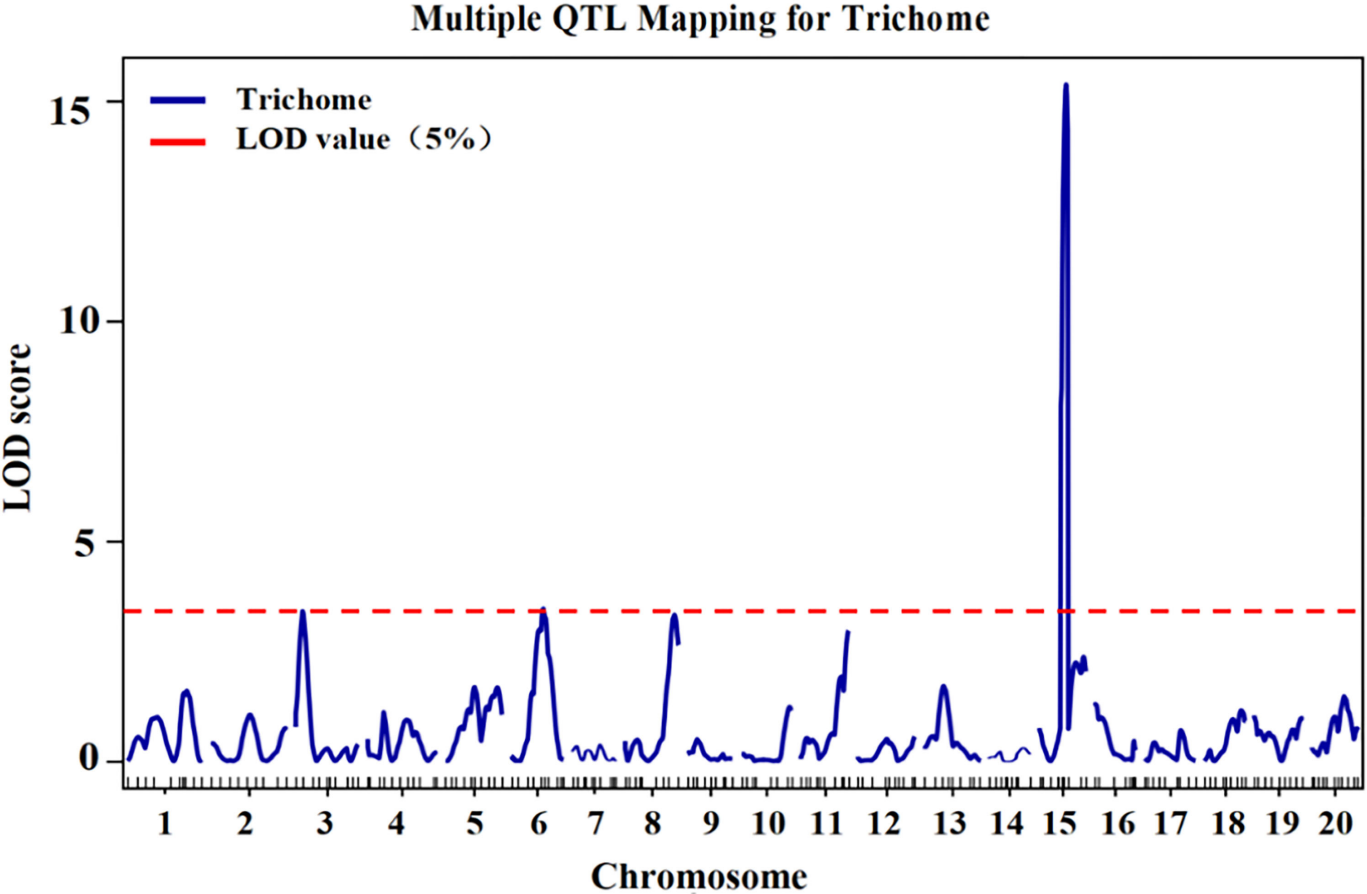
QTL mapping of trichome density by the genetic map in zucchini. According to the genotype and phenotype of the F2 population, three QTLs for trichome density were identified on chromosomes 3, 6, and 15. The *x*-axes indicate chromosomes, and the *y*-axes indicate logarithm of odds (LOD) score. The red horizontal lines indicate the LOD threshold value.

According to the genotype and phenotype of the F_2_ population, QTLs for trichome density were identified. Information on the QTLs was provided in **Table 1** and **Figure 3**. The LOD threshold was 3.43, and three QTLs were detected on chromosomes 3, 6, and 15. The major-effect QTL *CpTD15.1* was situated in a 1.54-Mb region between two markers, chr15-4229645 and chr15-5766791, and the peak marker was chr15-5766791. The LOD score of *CpTD15.1* was 15.38, explaining 36.74% of the observed phenotypic variation. The minor-effect QTL *CpTD3.1* was situated in a 3.16-Mb region between two markers, chr3-219017 and chr3-3381239, and the peak marker was chr3-1732969. The LOD score of *CpTD3.1* was 3.40, explaining 15% of the phenotypic variation. The minor-effect QTL *CpTD6.1* was situated in a 5.64-Mb region between two markers, chr6-2570712 and chr6-8212486, and the peak marker was chr6-5896890. The LOD score of *CpTD6.1* was 3.46, explaining 11.29% of the phenotypic variation.

**Table 1:** Mapping of QTLs for trichome density of type I and type II with genetic mapping.

QTL ID	Chr.	Start marker	End marker	Peak marker	Position intervals (Mb)	LOD	Add	Dom	PVE (%)
*CpTD3.1*	3	chr3-219017	chr3-3381239	chr3-1732969	3.16	3.40	0.49	0.24	15.00
*CpTD6.1*	6	chr6-2570712	chr6-8212486	chr6-5896890	5.64	3.46	−0.35	0.38	11.29
*CpTD15.1*	15	chr15-4229645	chr15-5766791	chr15-5766791	1.54	15.38	0.84	−0.36	36.74

PVE, phenotype variance explained; Add, additive effects; Dom, dominance effects.

### Stability analysis of QTLs with InDel markers

**Figure 4:**
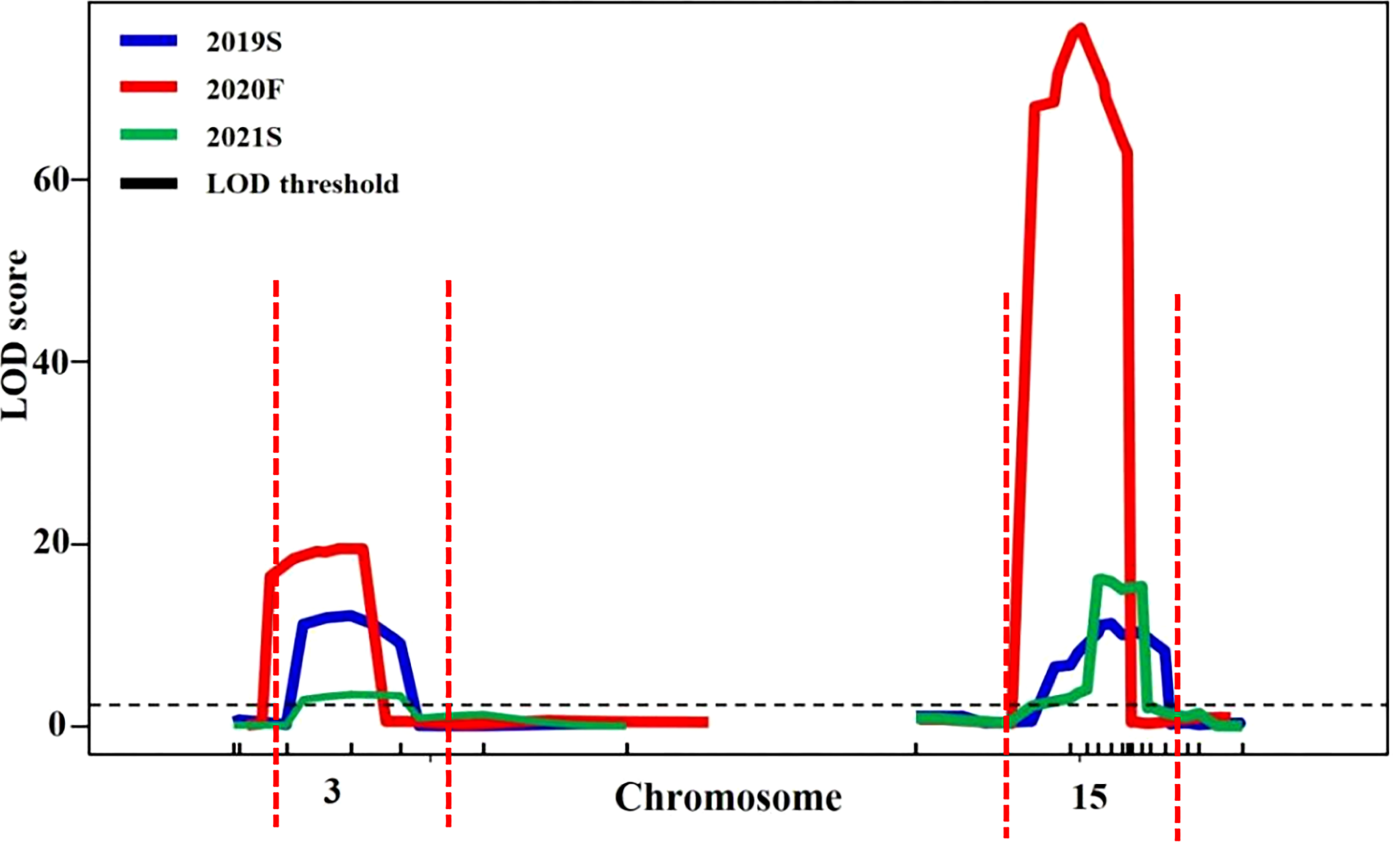
Stability analysis of *CpTD3.1* and *CpTD15.1* using F_2_ in three environments. The black horizontal line indicates the LOD = 3.0. According to data of three environments, the *CpTD3.1* locus was located in a 2,672.89-kb region by markers chr3-218350 and chr3-2891236, and the *CpTD15.1* locus was located in a 775.44-kb region by markers chr15-4991349 and chr15-5766791.

Candidate regions associated with trichome density were detected on chromosomes 3 and 15 by both QTL analysis methods. Therefore, polymorphic InDel markers on chromosomes 3 and 15 were developed to evaluate the stability of QTLs for trichome density. Plants of the F_2_ population grown under different environmental conditions were used for QTL mapping in R/qtl, and the genotype and phenotype of the F_2_ population are provided in **Table S8**. QTL starting markers, ending markers, peak markers, position intervals, LODs, PVEs, additive values, and dominance values are shown in **Table 2** and **Figure 4**. In 2019S, two QTLs, *CpTD3.1* and *CpTD15.1*, were identified. *CpTD3.1* was delimited by two markers, chr3-764774 and chr3-1732969, with a peak at chr3-1379216. *CpTD3.1* explained 10.64% of the observed phenotypic variation, *with* an LOD value of 12.10. *CpTD15.1* was delimited by two markers, chr15-5110786 and chr15-5766791, with a peak at chr15-5311576. *CpTD15.1* explained 12.71% of the phenotypic variation, with an LOD value of 11.93. In 2020F, two QTLs, *CpTD3.1* and *CpTD15.1*, were identified. *CpTD3.1* was delimited to the same region as *CpTD3.1* in 2019S (by two markers, chr3-218350 and chr3-1379216), with the same peak marker as *CpTD3.1* in 2019S*. CpTD3.1* explained 5.2% of the phenotypic variation, with an LOD value of 19.55. *CpTD15.1* was delimited to the same region as *CpTD15.1* in 2019S (by two markers, chr15-4991349 and chr15-5461456), with a peak at chr15-5224151. *CpTD15.1* explained 29.37% of the phenotypic variation, with an LOD value of 76.37. In 2021S, two QTLs, *CpTD3.1 and CpTD15.1*, were identified. *CpTD3.1* was delimited to the same region as *CpTD3.1* in 2019S and 2020F (by two markers, chr3-764774 and chr3-2891236), with the same peak marker as *CpTD3.1* in 2019S and 2020F*. CpTD3.1* explained 5% of the phenotypic variation, with an LOD value of 3.43. *CpTD15.1* was delimited to the same region as *CpTD15.1* in 2019S and 2020F (by two markers, chr15-5224151 and chr15-5619256), with the peak marker of *CpTD15.1* in 2019S*. CpTD15.1* explained 25.22% of the phenotypic variation, with an LOD value of 16.12.

**Table 2:** QTL analysis of *CpTD15.1* and *CpTD3.1* in different environmental conditions.

Environment	Chr.	QTL ID	Starting marker	Ending marker	Peak marker	Position intervals (kb)	LOD	Add	Dom	PVE (%)
2019S	3	*CpTD3.1*	chr3-764774	chr3-1732969	chr3-1379216	968.20	12.10	0.28	−0.10	10.64
	15	*CpTD15.1*	chr15-5110786	chr15-5766791	chr15-5311576	656.01	11.93	0.31	0.10	12.71
2020F	3	*CpTD3.1*	chr3-218350	chr3-1379216	chr3-1379216	1,160.87	19.55	0.27	0.03	5.20
	15	*CpTD15.1*	chr15-4991349	chr15-5461456	chr15-5224151	470.11	76.37	0.62	0.25	29.37
2021S	3	*CpTD3.1*	chr3-764774	chr3-2891236	chr3-1379216	2,126.47	3.43	0.28	−0.07	5.00
	15	*CpTD15.1*	chr15-5224151	chr15-5619256	chr15-5311576	395.11	16.12	0.65	0.31	25.22

PVE, phenotype variance explained; Add, additive effects; Dom, dominance effects.

**Figure 5:**
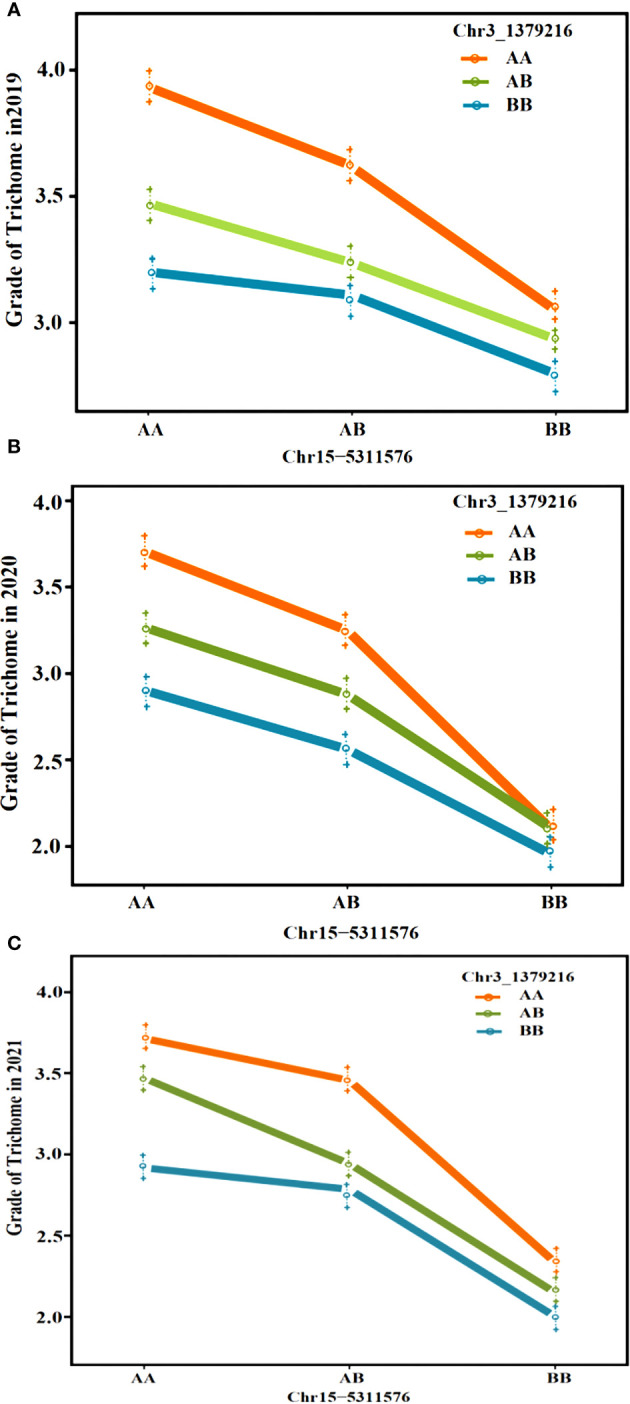
The genetic interaction analysis between *CpTD3.1* and *CpTD15.1*. There was a simple additive gene effect between *CpTD3.1* and *CpTD15.1*. **(A)** The genetic interaction analysis of two QTLs in 2019S. **(B)** The genetic interaction analysis of two QTLs in 2020F. **(C)** The genetic interaction analysis of two QTLs in 2021S.

Regarding the intersection of associated regions in different environments, the *CpTD3.1* locus was located in a 2,672.89-kb region based on the markers chr3-218350 and chr3-2891236, and the *CpTD15.1* locus was located in a 775.44-kb region based on the markers chr15-4991349 and chr15-5766791. *CpTD15.1* was *the* major-effect locus, and the region harbored candidate genes for trichome density. A genetic interaction analysis between *CpTD3.1* and *CpTD15.1* was performed and is illustrated in **Figure 5**. Typical parallels were observed across all genotypes indicating that there was no strong epistatic interaction between *CpTD3.1* and *CpTD15.1*. There was a simple additive gene effect between *CpTD3.1* and *CpTD15.1*.

### Candidate genes for trichome density

According to the Cucurbit Genomics Database, a total of 106 coding genes were located in the 775.44-kb region. To select the candidate genes associated with trichome density, gene structures and predicted functions in the region were analyzed. Five genes encoding GATA transcription factors, zinc finger proteins, and basic helix-loop helix (BHLH) transcription factors had the functions related to trichome development (**Table 3**). Three genes (*Cp4.1LG15g04030*, *Cp4.1LG15g04400*, and *Cp4.1LG15g04350*) showed nonsynonymous SNP/InDels in the CDS regions between “16” and “63” (**Figure S1**). Two genes (*Cp4.1LG15g04040* and *Cp4.1LG15g04400*) showed SNPs/InDels in the promoter elements between “16” and “63”, and one gene (*Cp4.1LG15g03820*) showed no SNPs/InDels in the CDS region or the promoter element. Among them, only *Cp4.1LG15g04400*, encoded ZFP gene, showed nonsynonymous SNPs in the conserved domain of the C3DHC3-type ring finger.

**Table 3:** Predicted genes in the major-effect QTL *CpTD15.1*.

Gene ID	Location	Promote element	Total/exon SNP and InDel	Type	Description
*Cp4.1LG15g03820*	5041268–5047572		0/0	Syn	GATA transcription factor
*Cp4.1LG15g04030*	5239496–5240203	–	7/2	Non-syn	Zinc finger-like protein
*Cp4.1LG15g04040*	5241709–5244982	Yes	9/0	Syn	Basic helix-loop helix transcription factor
*Cp4.1LG15g04400*	5557181–5567494	Yes	110/2	Non-syn	Zinc finger protein
*Cp4.1LG15g04350*	5569416–5570236	–	15/9	Non-syn	Zinc finger protein

Non-syn, non-synonymous; Syn, synonymous.

**Figure 6:**
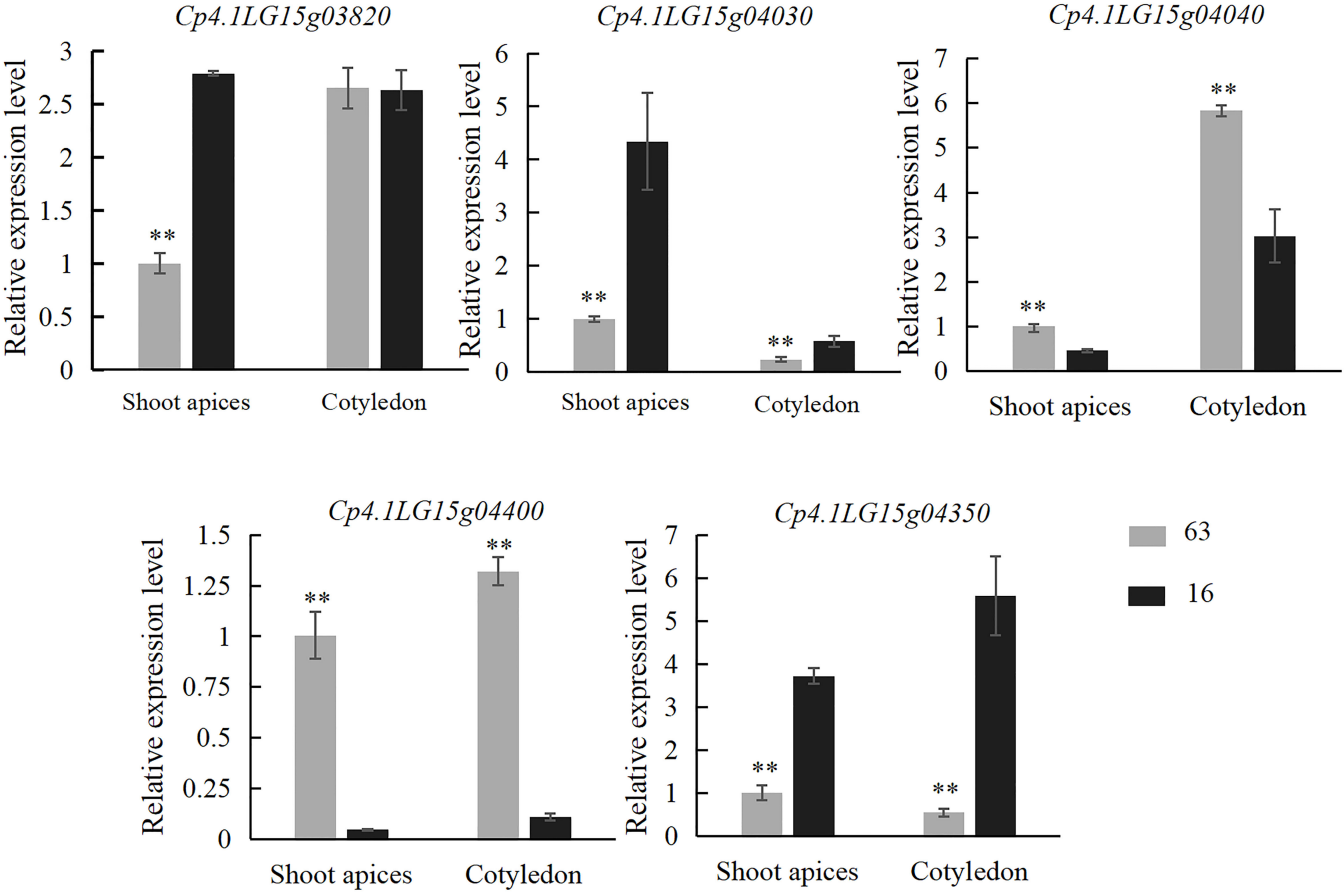
Relative expression levels of five candidate genes (*Cp4.1LG15g03820*, *Cp4.1LG15g04030*, *Cp4.1LG15g04040*, *Cp4.1LG15g04400*, and *Cp4.1LG15g04350*) in “16” and “63”. The relative expression levels of five genes were quantified using the 2^−ΔΔCT^ method. The expression level of the respective genes in leaf primordium of “16” was set to a value of 1 and used as a reference, respectively. Each was repeated three times. ** indicates an extremely significant difference, *p* < 0.01.

Trichomes from the third stage (the key stage for glandular and nonglandular trichome development) were collected from the base of cotyledon at 5 days after germination and leaf primordium at 7 days after germination on “16” and “63” plants. Expression analysis of all five genes was performed. The results showed that the expression levels of *Cp4.1LG15g04030* and *Cp4.1LG15g04350* in the SMT line “63” were significantly lower than those in the LDT line “16”, and the expression levels of *Cp4.1LG15g04040* and *Cp4.1LG15g04400 in the* SMT line “63” were significantly higher than those in the LDT line “16” (**Figure 6**). Zucchini germplasm resources consisting of five LDT lines and five SMT lines were selected, and leaf primordium of each line was collected for gene expression analysis. Only the *Cp4.1LG15g04400* gene showed significant expression differences between the LDT lines and SMT lines (**Figure S2**), and there was no significant difference in the expression of other four genes between the LDT lines and SMT lines. These results indicated that *Cp4.1LG15g04400* might be a good candidate gene for trichome density.

## Discussion

Trichomes are hairy structures covering aboveground tissues in terrestrial plants and provide an excellent model for studying cell differentiation and proliferation. Trichome development involves trichome density, trichome number, trichome distribution, trichome morphology, the production of secondary metabolic secretions, and so on. The R2R3 MYB, bHLH, HD-ZIP, and C2H2 ZFP families of transcription factors play significant roles in the regulation of trichome development in the model plants *Arabidopsis thaliana*, tomato, and cucumber. There are few reports on regulatory genes and trichome development mechanisms in zucchini. In our previous study, the multicellular trichome morphology of aboveground tissues of “16” and “63” was observed, and trichomes were classified into seven types: type I, II, III, and VII trichomes were nonglandular, and type IV, V, and VI trichomes were glandular. Type I and II trichomes were longer and harder than the other types, and the presence or absence of type I and II trichomes was an obvious difference between “16” and “63” (Lin et al., 2023). Type I and II trichomes of petioles were larger than those on other tissues and were dispersedly distributed on the surface; therefore, we could more easily observe trichome morphology and density by microscopy. As demonstrated by observing the density of type I and type II trichomes on petioles, the phenotypes of F_2_ plants were categorized into five classes. F_2_ individuals of class 1 had the sparsest type I and type II trichomes, and individuals of the class 5 phenotype had the densest type I and type II trichomes (Lin et al., 2023). In cucumber, the developmental processes involved in glandular and nonglandular multicellular trichome formation were observed using cotyledons as a model and divided into five sequential stages (Dong et al., 2022). In our study, zucchini cotyledons were used as a model and the same developmental processes of glandular and nonglandular trichomes were observed. The third stage (tip head formation/glandular head transition) was the key stage for glandular and nonglandular trichome development. The morphological observation and classification of zucchini trichomes laid a foundation for the QTL analysis of trichome density.

In our study, an F_2_ population derived by crossing “16” and “63” was constructed, and QTL analysis of the density of type I and type II trichomes was performed with QTL-seq and genetic map-based QTL analysis. Two QTLs associated with trichome density, *CpTD3.1* and *CpTD15.1*, were detected on chromosomes 3 and 15 by both QTL analysis methods. The stability of *CpTD3.1* and *CpTD15.1* was confirmed using data from three environmental conditions. The results showed that *CpTD3.1* and *CpTD15.1* were detected in similar regions in all environments, which explained 5%–10.64% and 12.71%–29.37% of the observed phenotypic variation, respectively. These two QTLs showed good stability, and the environment seldom affected the major-effect QTL *CpTD15.1* or the minor-effect QTL *CpTD3.1*. The *CpTD3.1* locus was within a 2,672.89-kb region between markers chr3-218350 and chr3-2891236, and the *CpTD15.1* locus was within a 775.44-kb region between markers chr15-4991349 and chr15-5766791. The genetic interaction analysis indicated that there was a simple additive gene effect between *CpTD3.1* and *CpTD15.1*. F_2_ individuals with the *CpTD15.1* locus derived from parent “16” and the *CpTD3.1* locus derived from parent “63” were labeled 15A-3B, individuals with the *CpTD15.1* locus derived from parent “63” and the *CpTD3.1* locus derived from parent “16” were labeled 15B-3A, and individuals with the *CpTD15.1* and *CpTD3.1* loci derived from parent “16” were labeled 15A-3A. The phenotypes of 15A-3B, 15B-3A, and 15A-3A in 2020F were collected. The trichome density of 15A-3B ranged from class 2 to class 4 with an average class of 2.86, the trichome density of 15B-3A ranged from class 1 to class 3 with an average class of 2.04, and the trichome density of 15A-3A ranged from 3 to 5 with an average class of 3.74 (**Figure S3**). The results confirmed that zucchini plants with both loci derived from parent “16” had dense trichomes. Conventional breeding techniques require the selection of phenotypes and evaluation in multiple environments for many years, and this process could require considerable time, labor, and land. Marker-assisted selection (MAS) is a powerful tool for selecting the target trait, which effectively accelerates the breeding process. Markers closely linked to the *CpTD15.1* and *CpTD3.1* loci (chr3-218350, chr3-2891236, chr15-4991349, and chr15-5766791) could potentially aid in the selection of trichome density at the seedling stage and be successfully used in MAS.

ZFPs are one of the most important transcription factors in plants. Based on the different number and arrangement of Cys and His residues, ZFPs can be categorized into nine types, namely, C2H2, C8, C6, C3HC4, C2HC, C2HC5, C4, C4HC3, and CCCH (Berg and Shi, 1996). ZFPs play vital roles in the regulation of growth, developmental processes, and multiple stress responses in plants (Bernhardt et al., 2003; Davletova et al., 2005; Dinneny et al., 2006; Xiao et al., 2009; Zhang et al., 2012). Many C2H2 ZFP genes have also been reported to be involved in trichome formation in unicellular or multicellular trichome plants (Davletova et al., 2005; Dinneny et al., 2006; Xiao et al., 2009; Zhang et al., 2012). In *Arabidopsis*, the first-identified C2H2 ZFP, GLABROUS INFLORESCENCE STEMS (GIS), induces trichome initiation in inflorescence organs. It acts upstream of the MYB-bHLH-WD complex and *GL1* by gibberellin (GA), and acts downstream of *SPINDLY* to promote inflorescence trichome initiation (Gan et al., 2006). Two C2H2 ZFPs, GIS2 and ZFP8, are integrated with cytokinin and GA signaling in the regulation of trichome initiation on inflorescence organs and cauline leaves, respectively (Gan et al., 2007). ZFP5, ZFP6, and GIS3 are the key proteins involved in trichome initiation on inflorescence organs, acting upstream of *GIS*, *GIS2*, and *ZFP8* (Zhou et al., 2012). In tomato, the *SlH*, *SlHair2/SlZFP8L*, and *SlZFP6* genes are members of the C2H2 family. *SlH* increases the length of type I, III, and VI trichomes. *SlH* and its closest homologue, *SlZFP8L*, physically interact with *Wo*, and SlH, zinc finger protein-like 8 (SlZFP8L), *and* Wo function together to regulate long-stalk type I trichome initiation (Chang et al., 2018; Chun et al., 2021). *SlZFP6* and *SlZFP8L* are critical for the density of type I, III/V, VI, and VII trichomes and increase the length of long-stalk type I, III, and VI trichomes. SlH interacts with SlZFP8-like to regulate trichome initiation and elongation by modulating *SlZFP6* gene expression. Based on transgenic technology, *SlH*, *SlZFP8L*, and *SlZFP6* may promote trichome elongation by activating the expression of cell-wall-loosening protein genes (Zheng et al., 2022). In conclusion, C2H2 zinc finger transcription factors play similar roles in the regulation of trichome formation in unicellular trichome plant *Arabidopsis and* multicellular trichome plant tomato. Cucumber fruits are covered with tubercules and spines. The tuberculate fruit gene *Tu*, which was identified as a C2H2 ZFP gene, is involved in the CTK biosynthetic pathway (Yang et al., 2014). In our research, genes encoding transcription factors were screened in the region of the major-effect locus *CpTD15.1*. Among these genes, one (*Cp4.1LG15g04400*) encoding ZFP harbored nonsynonymous SNPs in the conserved ring finger domain. The ring finger domain of *Cp4.1LG15g04400* was of the C3DHC3 type, which is found in *ZFPL1* genes. There were significant differences in *Cp4.1LG15g04400* expression between the LDT parent “16” and the SMT parent “63”, and a similar pattern was shown between zucchini LDT lines and SMT lines. A phylogenetic analysis of *Cp4.1LG15g04400* with known genes was performed, and *Cp4.1LG15g04400* was found to be a distant relative of C2H2 *ZFP* genes (**Figure S4**). Based on sequence analysis, gene function annotation, and gene expression analysis, it was presumed that *Cp4.1LG15g04400* might regulate trichome density as a new type of ZFP gene in zucchini. Near-isogenic lines of *CpTD15.1* were constructed to confirm the candidate gene of the major locus. The gene functions and regulatory pathways related to the trichome density of *Cp4.1LG15g04400* need to be further validated based on more molecular biological experiments in zucchini.

## Conclusion

An F_2_ population was constructed with the LDT inbred line “16” and the SMT inbred line “63” for QTL analysis of type I and II trichome density. Two QTLs, *CpTD3.1 and CpTD15.1*, were identified on chromosomes 3 and 15 using the QTL-seq method and genetic map-based QTL analysis, and *CpTD15.1* was the major-effect QTL. The stability of *CpTD3.1* and *CpTD15.1* was confirmed using data from F_2_ plants under different environmental conditions. The major-effect QTL *CpTD15.1* was located between markers chr15-4991349 and chr15-5766791 with a physical distance of 775.44 kb, and explained 12.71%–29.37% of the phenotypic variation observed in the three environments. *CpTD3.1* was located between markers chr3-218350 and chr3-2891236, with a physical distance of 2,672.89 kb, and explained 5.00%–10.64% of the phenotypic variation in the three environments. In the *CpTD15.1* region, five genes encoding transcription factors regulating trichome development were selected. *Cp4.1LG15g04400* harbored nonsynonymous SNPs in the conserved ring finger domain between the two parental lines. There were significant differences in *Cp4.1LG15g04400* expression between “16” and “63” and in the germplasm resources of LDT lines and SMT lines. It was inferred that *Cp4.1LG15g04400* might regulate trichome density in zucchini. This study lays a foundation for better understanding the density of multicellular nonglandular trichomes and the regulatory mechanism of trichome density in zucchini.

## Data availability statement

The datasets presented in this study can be found in online repositories. The names of the repository/repositories and accession number(s) can be found below: BioProject accession number: PRJNA987358.

## Author contributions

YW performed data analysis and prepared the manuscript. GW contributed to RNA extraction and qRT-PCR test. DL contributed to collecting phenotypic characteristics and DNA extraction. QL contributed to growing plants. WX and SQ, the corresponding authors, oversaw all activities related to the project implementation and manuscript development. All authors read and approved the final version of the manuscript.

## Supplementary material

Supplementary Figure 1 Alignment of the predicted protein sequences of five genes between “16” and “63”. **(A)** Alignment acid sequences of *Cp4.1LG15g04030*. **(B)** Alignment acid sequences of *Cp4.1LG15g04400*. **(C)** Alignment acid sequences of *Cp4.1LG15g04350*. The red lines indicated ring finger domains.

Supplementary Figure 2 Alignment of the predicted protein sequences of five genes between germplasm resources of LDT lines and SMT lines. The relative expression levels of five genes were quantified using the 2^−ΔΔCT^ method. The expression level of the respective genes in the first LDT line was set to a value of 1 and used as a reference, respectively. Each was repeated three times. Capital letters indicate an extremely significant difference, *p* < 0.01.

Supplementary Figure 3 Trichome density of 15A-3B, 15B-3A, and 15A-3A in F_2_. Capital letters indicate an extremely significant difference, *p* < 0.01.

Supplementary Figure 4 Phylogenetic tree analysis of Cp4.1LG15g04400 and known ZFPs.
