# Supplementary material for: QTL mapping and stability analysis of trichome density in zucchini (Cucurbita pepo L.)
Source: Front Plant Sci. 2023 Aug 11;14:1232154. doi: 10.3389/fpls.2023.1232154 (PMC10457680; doi:10.3389/fpls.2023.1232154)
Supplement: Supplementary file 2 [file Table_2.docx]

Table S2. InDel markers on chromosomes 3 and 15

| Marker name | Primer sequence (5′–3′) | Length (bp) | Tm (℃) |
| --- | --- | --- | --- |
| chr15_3394422 | F: CCGCTTGCCAAAATAAC  R: GTCACCCACCCTCACTATG | 156/178 | 57 |
| chr15_4991349 | F: GCAAGAAACAAAGGTGGGA  R: GTATGTGGTGTAGGGCGG | 219/229 | 57 |
| chr15_5110786 | F: TGGCACCCAATCACAATAG  R: CTGTTTGGAGAAGGAGCGTA | 123/122 | 57 |
| chr15_5224151 | F: AGCCATCGTGTGTATTTTCCC  R: AACAACCTGGTGGGGTGATT | 298/330 | 57 |
| chr15_5311576 | F: CTTCGGGGCTCAGACTC  R: AACTACTCCACAACTCAACCT | 288/326 | 56 |
| chr15_5461456 | F: CGTTCCTCACAACCCTTC  R: GTCTATGGTTTCTGATGCTTCT | 159/180 | 57 |
| chr15_5513468 | F: TTATGGATTGGGACTTGGC  R: TTTGGGACGGCTTTTTTG | 174/186 | 56 |
| chr15_5582399 | F: AGAGTAGGTGACAGGCAAGATT  R: AACCCAAAGAGAAGAGCACTG | 160/173 | 58 |
| chr15_5619256 | F: ATCAAAAGTCCCAAACAAGA  R: TGATGGCTTAGATTTCTTCCT | 143/152 | 56 |
| chr15_5672054 | F: TTGAGAAGATGGCGATGAGTATG  R: CGAGAATGAGTCCTACGTTAGC | 261/283 | 59 |
| chr15_5766791 | F: CTTTGTCGGTCCCGTGAG  R: TCGTTTAGGGTTTATGGGTTC | 224/236 | 56 |
| chr15_5827733 | F: TCCTACCCCGTATCCACT  R: TCTATTCCTTGCTACCCTTC | 167/207 | 56 |
| chr15_5897945 | F: CGAGTGGCTGACGAGGAC  R: CCAGAGGTTGGAGGAGAATAA | 200/210 | 57 |
| chr15_6184888 | F: AACACAATCCGTTCGTCCA  R: TGGAATGCCTTCTTGTAACG | 133/150 | 55 |
| chr3_147831 | F: CAAAAGTAATGGTAGTGGGG  R: ATTATCATCGTATGGCAAGA | 232/255 | 52 |
| chr3_218350 | F: ATTTCAGGAGTTGGGCG  R: TTAGCGGGTGACAGTGG | 117/128 | 56 |
| chr3_764774 | F: TGGGATTGTGTCTTTGGC  R: TTGTCTGCTTTGGGCTTT | 148/168 | 54 |
| chr3_1379216 | F: ACCATTGGAGGTGATGTATTG  R: TTCAGTGGTCCGTTACTTTCA | 251/274 | 55 |
| chr3_1732969 | F: CCACCCACGAGCCAAGTA  R: GGAAAGGGAGCGGACAAC | 180/198 | 58 |
| chr3_2472555 | F: AGATTTTAGTCGGTCGCTCC  R: TTCTTTCCTTTCAGGGCAAC | 204/253 | 55 |
| chr3_2891236 | F: AGCCACCTCAGAAGATGCA  R: TTGTAAGGACCAAACCCATT | 146/155 | 56 |
