# Supplementary material for: QTL mapping and stability analysis of trichome density in zucchini (Cucurbita pepo L.)
Source: Front Plant Sci. 2023 Aug 11;14:1232154. doi: 10.3389/fpls.2023.1232154 (PMC10457680; doi:10.3389/fpls.2023.1232154)
Supplement: Supplementary file 3 [file Table_3.docx]

Table S3. Primers for qRT-PCR

| Primer name | Primer sequence (5′–3′) | Length |
| --- | --- | --- |
| *Cp4.1LG15g03370* | F: GGTTGGAGTTGTCAATCGTTC  R: TTGTCAGCCAAGTGTCTGTAGTC | 120 bp |
| *Cp4.1LG15g03820* | F: GACTCCAATTTCCAAGACGC  R: AAGGGTTGTAATCGGAGCC | 223 bp |
| *Cp4.1LG15g04030* | F: GATACGAAAGCCTGGTGGTG  R: CTGGATTAGTCGTCGTTCGC | 161 bp |
| *Cp4.1LG15g04040* | F: TCTGAGATGGGAATGGAGGAC  R: GATACGAACTCCATCCATCACTC | 214bp |
| *Cp4.1LG15g04350* | F: TCCTCAATCATAAGCCCTTCG  R: AGACACGGTGGCAGTAATCG | 230 bp |
| *Cp4.1LG15g04400* | F: TCAATAGCAAACATCGAAACG  R: GGACCTCTTCGAACATACTTCTTAC | 260 bp |
