# Supplementary material for: QTL mapping and stability analysis of trichome density in zucchini (Cucurbita pepo L.)
Source: Front Plant Sci. 2023 Aug 11;14:1232154. doi: 10.3389/fpls.2023.1232154 (PMC10457680; doi:10.3389/fpls.2023.1232154)
Supplement: Supplementary file 4 [file Table_4.docx]

Table S4. Summary of genome resequencing data

| Sample | Clean reads | Clean base | Average depth | Coverage ratio 1× (%) | Q30(%) | GC(%) | Mapped(%) |
| --- | --- | --- | --- | --- | --- | --- | --- |
| 16 | 27,795,367 | 8,327,060,892 | 17 | 93.99 | 91.37 | 38.65 | 97.85 |
| 63 | 41,317,788 | 12,378,579,886 | 24 | 95.72 | 91.72 | 39.10 | 98.23 |
| D-pool | 47,285,715 | 14,166,380,226 | 28 | 97.22 | 92.42 | 38.94 | 98.07 |
| S-pool | 38,480,663 | 11,525,228,208 | 23 | 97.14 | 91.49 | 38.79 | 97.96 |
