# Supplementary material for: QTL mapping and stability analysis of trichome density in zucchini (Cucurbita pepo L.)
Source: Front Plant Sci. 2023 Aug 11;14:1232154. doi: 10.3389/fpls.2023.1232154 (PMC10457680; doi:10.3389/fpls.2023.1232154)
Supplement: Supplementary file 5 [file Table_5.docx]

Table S5. QTL mapping for trichome density of type I and type II with QTL-seq

| Method | Chromosome | Position (Mb) | Size (Mb) | Gene number |
| --- | --- | --- | --- | --- |
| ED-SNP | 3 | 0-2.70 | 2.70 | 466 |
|  | 15 | 3.65-8.65 | 5.00 | 607 |
| ED-InDel | 3 | 0-3.00 | 3.00 | 509 |
|  | 15 | 3.68-8.66 | 4.98 | 605 |
| SNP-index | 15 | 4.97-6.30 | 1.33 | 171 |
| InDel-index | 15 | 5.06-6.17 | 1.11 | 144 |
| Total | 3 | 0-3.00 | 3.00 | 509 |
|  | 15 | 3.65-8.65 | 5.00 | 607 |
