# Supplementary material for: QTL mapping and stability analysis of trichome density in zucchini (Cucurbita pepo L.)
Source: Front Plant Sci. 2023 Aug 11;14:1232154. doi: 10.3389/fpls.2023.1232154 (PMC10457680; doi:10.3389/fpls.2023.1232154)
Supplement: Supplementary file 7 [file Table_7.docx]

Table S7. Genetic linkage map with InDel markers

| Chromosome | Marker | Map length (cM) | Marker interval  (cM) | Spearman | Chromosome | Marker | Map length (cM) | Marker interval  (cM) | Spearman |
| --- | --- | --- | --- | --- | --- | --- | --- | --- | --- |
| 1 | 10 | 194.92 | 19.49 | 0.99 | 11 | 10 | 125.46 | 12.55 | 0.99 |
| 2 | 9 | 197.94 | 22.00 | 0.99 | 12 | 10 | 149.36 | 154.94 | 0.99 |
| 3 | 10 | 164.47 | 16.45 | 1.00 | 13 | 9 | 145.96 | 16.22 | 0.99 |
| 4 | 11 | 179.92 | 16.36 | 1.00 | 14 | 9 | 107.91 | 11.99 | 1.00 |
| 5 | 10 | 148.93 | 14.89 | 1.00 | 15 | 10 | 121.86 | 12.19 | 1.00 |
| 6 | 10 | 137.02 | 13.70 | 0.99 | 16 | 8 | 104.97 | 13.12 | 0.98 |
| 7 | 10 | 112.46 | 11.25 | 0.99 | 17 | 9 | 133.26 | 14.81 | 1.00 |
| 8 | 10 | 140.43 | 14.04 | 0.99 | 18 | 9 | 106.16 | 11.80 | 1.00 |
| 9 | 9 | 116.64 | 12.96 | 0.99 | 19 | 9 | 126.58 | 14.06 | 1.00 |
| 10 | 8 | 131.08 | 16.39 | 0.99 | 20 | 11 | 120.32 | 10.94 | 1.00 |
|  |  |  |  |  | Sum | 191 | 2765.65 | 14.48 | 0.99 |
