# Supplementary figures and images for: QTL mapping and stability analysis of trichome density in zucchini (Cucurbita pepo L.)
Source: Front Plant Sci. 2023 Aug 11;14:1232154. doi: 10.3389/fpls.2023.1232154 (PMC10457680; doi:10.3389/fpls.2023.1232154)

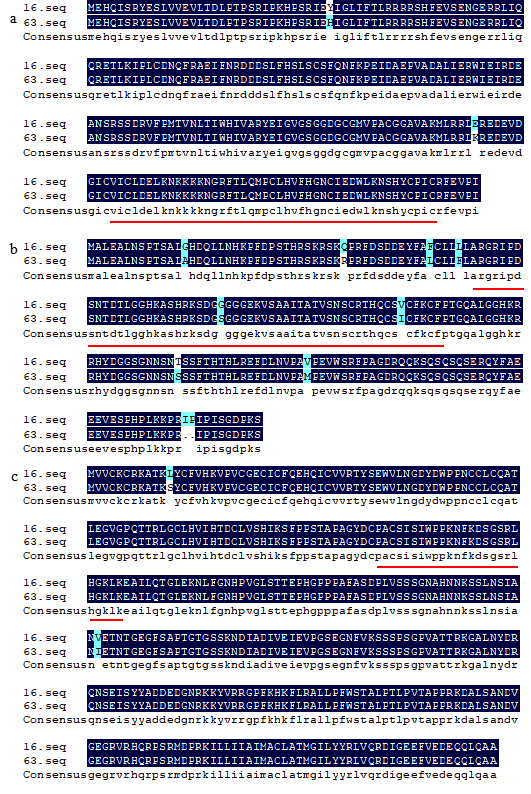

Supplement: Supplementary Figure 1 — Alignment of the predicted protein sequences of five genes between “16” and “63”. (A) Alignment acid sequences of Cp4.1LG15g04030. (B) Alignment acid sequences of Cp4.1LG15g04400. (C) Alignment acid sequences of Cp4.1LG15g04350. The red lines indicated ring finger domains. [file Image_1.png]

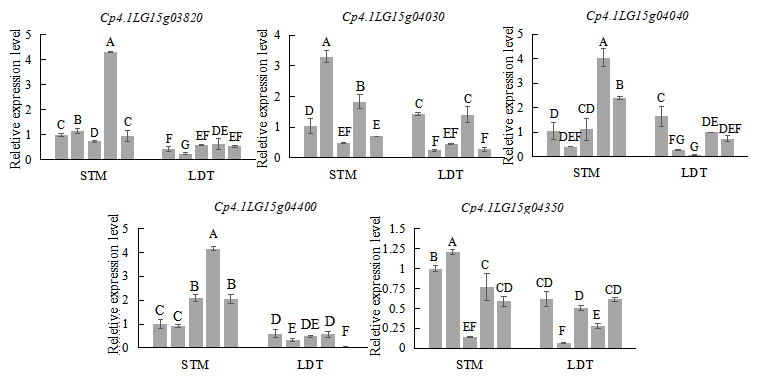

Supplement: Supplementary Figure 2 — Alignment of the predicted protein sequences of five genes between germplasm resources of LDT lines and SMT lines. The relative expression levels of five genes were quantified using the 2−ΔΔCT method. The expression level of the respective genes in the first LDT line was set to a value of 1 and used as a reference, respectively. Each was repeated three times. Capital letters indicate an extremely significant difference, p < 0.01. [file Image_2.png]

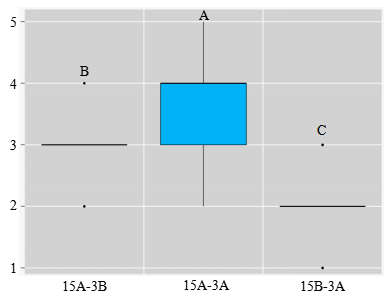

Supplement: Supplementary Figure 3 — Trichome density of 15A-3B, 15B-3A, and 15A-3A in F2. Capital letters indicate an extremely significant difference, p < 0.01. [file Image_3.png]

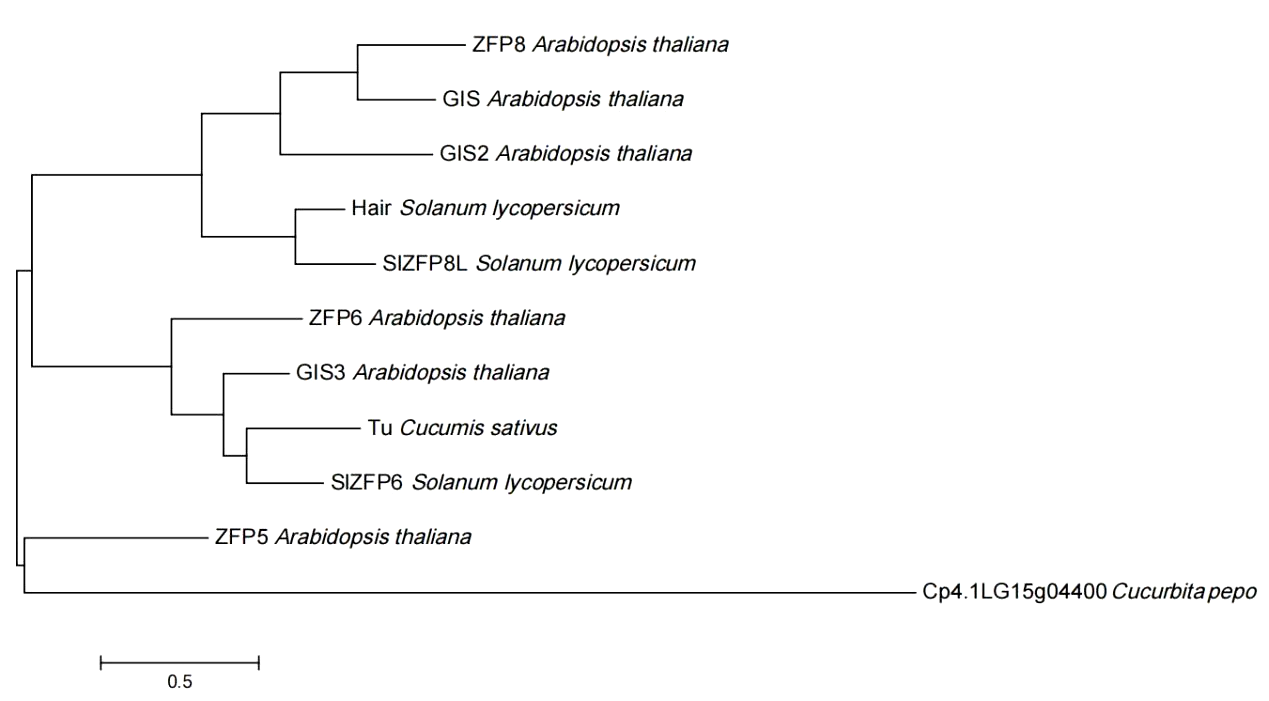

Supplement: Supplementary Figure 4 — Phylogenetic tree analysis of Cp4.1LG15g04400 and known ZFPs. [file Image_4.png]
